# Supplementary figures and images for: ANRIL regulates multiple molecules of pathogenetic significance in diabetic nephropathy
Source: PLoS One. 2022 Aug 19;17(8):e0270287. doi: 10.1371/journal.pone.0270287 (PMC9390929; doi:10.1371/journal.pone.0270287)

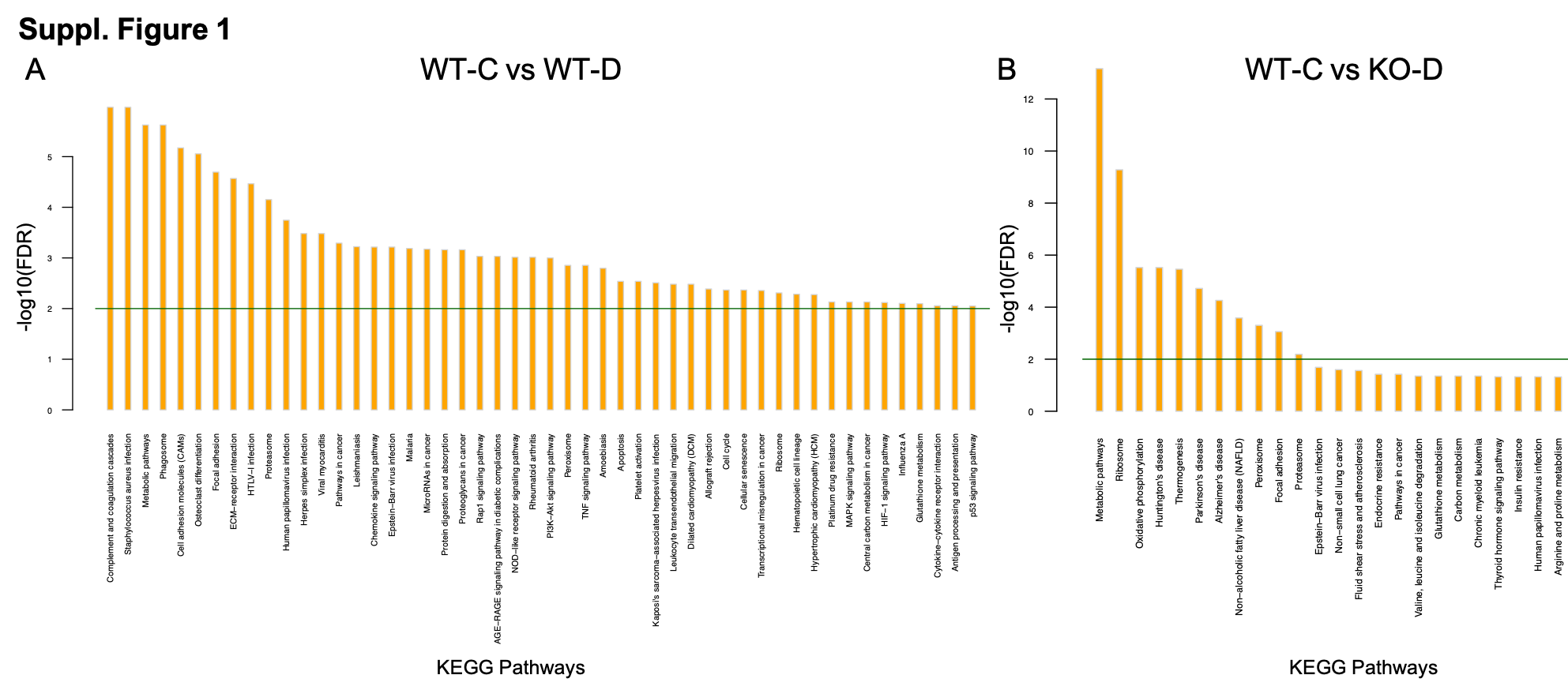

Supplement: S1 Fig — KEGG pathway analyses showed alterations (p<0.05) of transcripts related to multiple biological pathways in the kidneys in diabetes (A). The majority of these pathways were corrected in the ANRIL KO mice (B). (C = non-diabetic control, D = poorly controlled diabetic, WT = wild type, KO = ANRIL knockout. Horizontal line shows false discover rate of 0.01, the analyses for differentially expressed genes at the level of P<0.01 have been depicted in the Fig 3A. The detailed listings of these transcripts are in the supplementary tables (S1 and S2 Tables). (TIF) [file pone.0270287.s001.tif]

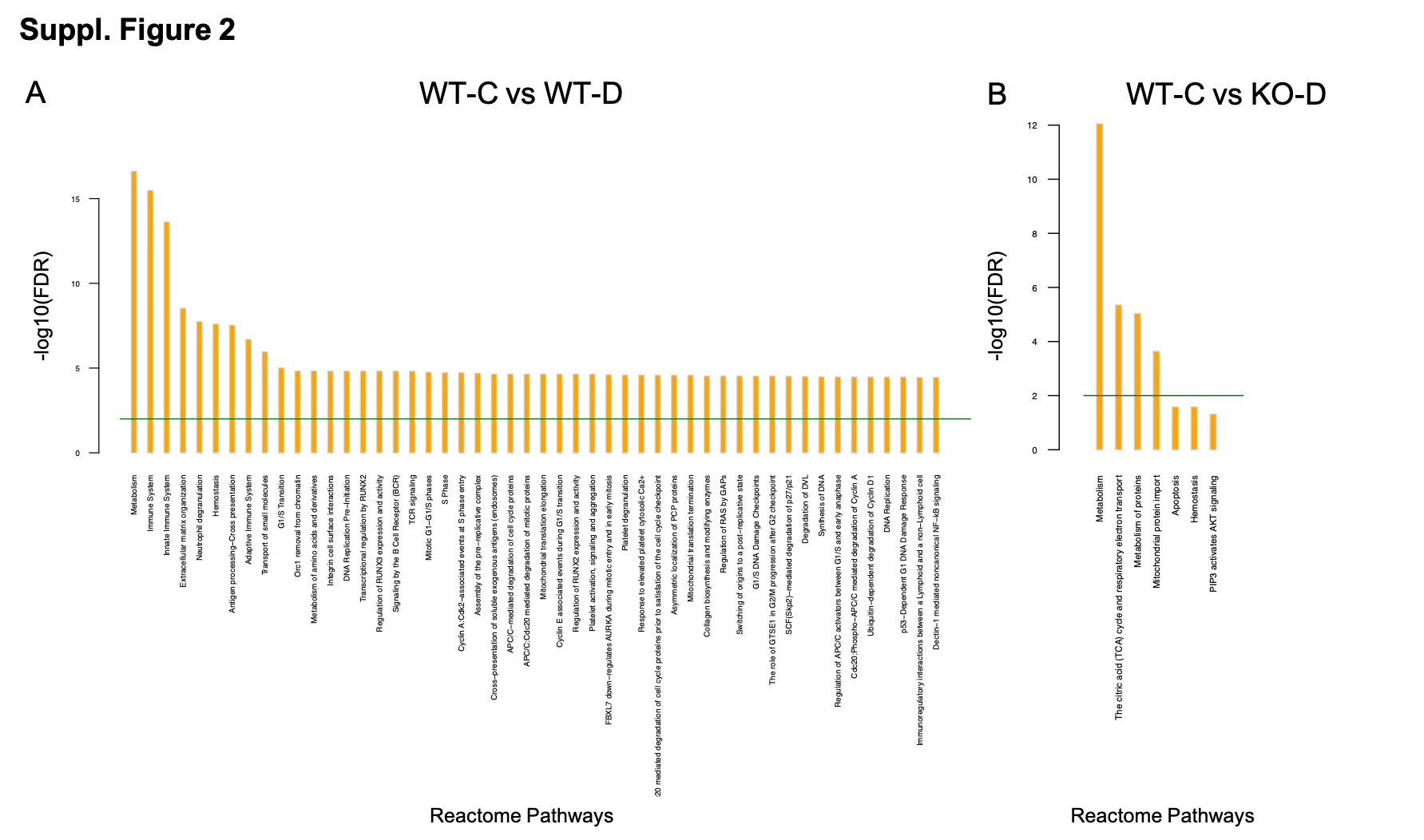

Supplement: S2 Fig — A) Reactome pathway analyses showed alterations (p<0.05) of transcripts related to multiple biological pathways in the kidneys in diabetes. (A). The majority of these pathways were corrected in the ANRIL KO mice (B). (C = non-diabetic control, D = poorly controlled diabetic, WT = wild type, KO = ANRIL knockout. Horizontal line shows false discover rate of 0.01, the analyses for differentially expressed genes at the level of P<0.01 have been depicted in the Fig 3E. The detailed listings of these transcripts are in the supplementary tables (S1 and S2 Tables). (TIF) [file pone.0270287.s002.tif]
