# Supplementary material for: ANRIL regulates multiple molecules of pathogenetic significance in diabetic nephropathy
Source: PLoS One. 2022 Aug 19;17(8):e0270287. doi: 10.1371/journal.pone.0270287 (PMC9390929; doi:10.1371/journal.pone.0270287)
Supplement: Attachment — Submitted filename: Review comments.docx [file pone.0270287.s006.docx]

In this study, Sooshtari et al. identified long non-coding RNA ANRIL, was upregulated, and plays a significant role in pathogenesis of diabetic nephropathy.

This study is original and interesting. The manuscript is well written, data are convincing, well presented. There are few areas needs to clarify,

1) Since there multiple non-coding RNAs involved in diabetic pathogenies, what is rational behind choosing lncRNA ANRIL?
2) Why author didn’t quantify Haemoglobin A_1c_ (HbA_1c_) levels after mice induced with streptozotocin, did you find any difference in HA1c between WT-D vs KO-D cohort?
3) Does ANRIL-KO make any other complications in mice? Since, this KO alter multiple gene functions? And it plays functional role in pathogenesis of diabetic nephropathy
4) The study extensively did an in-silico approach for reporting functional regulation of genes, metabolic pathway alteration and protein interaction altered by ANRIL-KO, it would be more evidential, if it is confirmed with some real time experiments
5) What is reason behind using two different pathway analyzer KEGG and Reactome for pathway analysis?
6) Author identified an interesting factor that normal vs ANRIL-KO mice has no significant alterations in basal gene expression, what makes the significant difference in gene transcriptional level upon diabetic induced mice?
7) Did author generated any new data set from RNA sequencing between WT-D vs ANRIL-KO-D, if so then you didn’t submit to gene bank? If submitted what is the accession ID?

Limitation of the study is in-silico bioinformatic approach
