# Supplementary material for: ANRIL regulates multiple molecules of pathogenetic significance in diabetic nephropathy
Source: PLoS One. 2022 Aug 19;17(8):e0270287. doi: 10.1371/journal.pone.0270287 (PMC9390929; doi:10.1371/journal.pone.0270287)
Supplement: Attachment — Submitted filename: Comments_PONE-D-21-38242.docx [file pone.0270287.s007.docx]

“ANRIL regulates multiple molecules of pathogenetic significance in diabetic Nephropathy”

Diabetes is the most common cause of ESRD, and thus molecular mechanisms in DN need to explore more to control the progression of DN. Epigenetic modification plays a crucial role in physiological and pathological conditions. The authors previously reported that lncRNA ANRIL arbitrated diabetes-associated abnormalities in DN using the ANRIL KO mice model. In this MS, the author addressed the downstream mechanisms of ANRIL alteration in DN based on RNA seq analysis using computational methods. They observed that ANRIL regulates multiple DN regulated transcripts. It is an expected observation, as the authors previously showed that ANRIL KO mice are protected from DN. Using protein-protein interaction and cluster analysis, they found a few non-characterized molecules, including known pathogenic proteins in the context of DN.

1. How many uncharacterized molecules are identified? Provide a list of molecules so that people can work in the future.
2. Does ANRIL regulate only DN-associated genes? How do you exclude other gens or uncharacterized genes in the context of DN?
3. ‘However, in both such analyses, minimal number of alterations of transcripts were seen at the basal level (WT-C vs KO-C), suggesting a minimal impact of these knockout on the downstream transcript at the basal level.’

Does ANRIL KO show any pre-programming by altering the DN-associated genes to protect diabetic-associated nephropathy? Are there have any differential expression genes in ANRIL KO compared to control (WT-C) in the context of DN?

4. Provide differentially expressed gene lists for each combination.
5. Does ANRIL regulate DN by targeting only DN-associated genes?

Minor revision.

1. The whole MS contains several track changes.
2. ‘Renal cortices were dissected out from the kidneys and kept frozen at 80oC for further’ It would be negative 80.

Overall, the authors analyzed RNA Seq data and used a bioinformatics approach to find the ANRIL mediated gene regulation. They did not prove any direct evidence that ANRIL modulates genes to protect DN. Still, this analysis would be a good source for future studies towards diabetes and diabetes-associated nephropathy.
