# Supplementary material for: ANRIL regulates multiple molecules of pathogenetic significance in diabetic nephropathy
Source: PLoS One. 2022 Aug 19;17(8):e0270287. doi: 10.1371/journal.pone.0270287 (PMC9390929; doi:10.1371/journal.pone.0270287)
Supplement: Attachment — Submitted filename: Reviewers comment- PONE-D-21-38242.docx [file pone.0270287.s008.docx]

**Reviewer’s comment**

Long non-coding RNA ANRIL is associated with coronary artery diseases, cancer, and diabetes. ANRIL is reported to have a role in diabetic kidney disease. In the study titled ‘ANRIL regulates multiple molecules of pathogenetic significance in diabetic nephropathy’ the authors Sooshtari et al have compared RNA expression in renal cortical tissues of ANRIL knockout (KO) mice and wild type (WT) mice, with or without streptozotocin (STZ) induced diabetes. Diabetic animals showed hyperglycemia, reduced body weight gain, polyuria, and increased urinary albumin. ANRIL knockout (KO) mice with and without STZ have corrected the polyuria and increased urinary albumin.

Differentially expressed genes were identified using edgeR and DESeq2. KEGG and Reactome pathway analyses and network analyses were done using STRING and IPA. Only a few genes were differentially expressed in control ANRIL knockout mice compared to control WT mice. Many genes were differentially expressed in Diabetic WT mice as compared to normal WT mice, and these genes belong to metabolic pathways, apoptosis, extracellular matrix protein synthesis and degradation, NFKB related pathways, AGE-RAGE interaction pathways, etc. ANRIL KO prevented most of these pathways.

This is an interesting study. The manuscript is well written, but the discussion part needs to be improved. Here are some suggestions which might improve the value of the article.

1. As per the manuscript, the study aims to identify the downstream mechanism of ANRIL in diabetic nephropathy. However, a detailed discussion on specific pathways of ANRIL is missing. Although the RNA expression varies with tissue type, if possible, the authors can find out the fate of known targets of ANRIL (Yin et al. 2021 PMID 33528317, Aarabi et al. 2018 PMID 29868613, Regmi et al. 2019 PMID 30835718, Ignarski et al. 2019 PMID 31277300, Dai et al. 2020 PMID 32256207) and include them in the discussion part too.
2. Check the table number and legends of Supplementary Table 2 and Supplementary Table 3
3. The authors have narrowed it down to some of the target genes of ANRIL. If possible, the authors can demonstrate changes in protein expression/phosphorylation of ANRIL targets in the tissues of KO mice or kidney cell lines by knocking down the ANRIL.
4. Is ANRIL regulates its targets through miRNA?
